# Bis[4-(4-amino­phenyl­sulfanyl)phenyl] ketone

**DOI:** 10.1107/S1600536809016948

**Published:** 2009-05-14

**Authors:** Gang Zhang, Yan-lun Wang, Sheng-ru Long, Jie Yang

**Affiliations:** a College of Chemistry, Sichuan University, Chengdu 610064, People’s Republic of China; b Analysis & Testing Centre, Sichuan University, Chengdu 610064, People’s Republic of China; c Institute of Materials Science & Technology, Sichuan University, Chengdu 610064, People’s Republic of China

## Abstract

The mol­ecule of the title compound, C_25_H_20_N_2_OS_2_, has imposed twofold rotation symmetry. The dihedral angle formed by the two crystallographically independent phenyl rings is 79.23 (7)°. In the crystal packing, the mol­ecules are linked by inter­molecular N—H⋯O hydrogen bonds, forming chains running parallel to [102].

## Related literature

For the properties and applications of the title compound and related derivatives, see: Wang *et al.* (2006*a* ▶,*b* ▶); Jiang *et al.* (2006 ▶); Aritomi & Terauchi (1985 ▶); Aritomi & Fujiwara (1986 ▶). For the synthesis of the title compound, see: Yang *et al.* (2007 ▶); Chen *et al.* (2009 ▶).
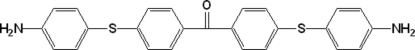


## Experimental

### 

#### Crystal data

C_25_H_20_N_2_OS_2_
*M* *_r_* = 428.55
Monoclinic, 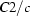

*a* = 18.945 (3) Å
*b* = 6.025 (2) Å
*c* = 20.793 (5) Å
β = 110.64 (4)°
*V* = 2221.1 (11) Å^3^
*Z* = 4
Mo *K*α radiation
μ = 0.26 mm^−1^
*T* = 292 K
0.52 × 0.48 × 0.42 mm

#### Data collection

Enraf–Nonius CAD-4 diffractometer
Absorption correction: spherical (*WinGX*; Farrugia, 1999 ▶) *T*_min_ = 0.877, *T*_max_ = 0.899
2261 measured reflections
1990 independent reflections
1441 reflections with *I* > 2σ(*I*)
*R*_int_ = 0.010
3 standard reflections every 150 reflections intensity decay: 2.4%

#### Refinement

*R*[*F* ^2^ > 2σ(*F* ^2^)] = 0.050
*wR*(*F* ^2^) = 0.146
*S* = 1.05
1990 reflections
145 parameters
H atoms treated by a mixture of independent and constrained refinement
Δρ_max_ = 0.22 e Å^−3^
Δρ_min_ = −0.30 e Å^−3^

### 

Data collection: *DIFRAC* (Gabe *et al.*, 1993 ▶); cell refinement: *DIFRAC*; data reduction: *NRCVAX* (Gabe *et al.*, 1989 ▶); program(s) used to solve structure: *SHELXS97* (Sheldrick, 2008 ▶); program(s) used to refine structure: *SHELXL97* (Sheldrick, 2008 ▶); molecular graphics: *ORTEP-3 for Windows* (Farrugia, 1997 ▶); software used to prepare material for publication: *SHELXL97*.

## Supplementary Material

Crystal structure: contains datablocks global, I. DOI: 10.1107/S1600536809016948/rz2311sup1.cif

Structure factors: contains datablocks I. DOI: 10.1107/S1600536809016948/rz2311Isup2.hkl

Additional supplementary materials:  crystallographic information; 3D view; checkCIF report

## Figures and Tables

**Table 1:** Hydrogen-bond geometry (Å, °)

*D*—H⋯*A*	*D*—H	H⋯*A*	*D*⋯*A*	*D*—H⋯*A*
N1—H*N*2⋯O1^i^	0.77 (3)	2.52 (3)	3.231 (4)	154 (3)

Symmetry code: (i) 
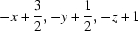
.

**Fig. 1.**
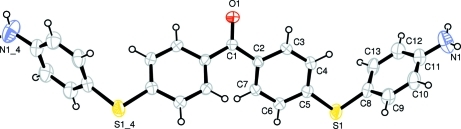
The molecular structure of the title compound. Unlabelled atoms are related to the labelled atoms by the symmetry operator (1-x, y, 1/2-z).

## supplementary crystallographic information

### Comment

The title compound is a major active photo-initiator used in coatings, optics and microelectronic materials (Wang *et al.*, 2006a,b; Jiang *et al.*, 2006) and can be used as monomer in the synthesis of high performance polyamide. Moreover, as photo initiator it has showed superior to the natural compound 4,4'-difluorobenzophenone (Wang *et al.*, 2006a). Besides their properties as photo-initiators, some derivatives of the title compound have also been reported to possess good thermostability and chemical resistance (Aritomi & Terauchi, 1985; Aritomi & Fujiwara, 1986). The synthetic procedure of the title compound have been reported elsewhere (Yang *et al.*, 2007; Chen *et al.*, 2009).

The molecule of the title compound (Fig. 1) has crystallographically imposed twofold rotation symmetry. In the asymmetric unit, the phenyl rings form a dihedral angle of 79.23 (7)°. The C2–C1–C2^i^–C7^i^ torsion angle is 29.49 (15)° (symmetry code: i = 1-x, y, 1/2-z). In the crystal packing, intermolecular N—H···O hydrogen bonding interactions (Table 1) link the molecules into chains running parallel to the [102] direction.

### Experimental

A mixture of 4,4'-difluorobenzophenone (21.8 g, 0.1 mol), 4-aminothiophenol (25 g, 0.2 mol), K_2_CO_3_ (14.0 g, 0.101 mol) and dimethyl acetamide (120 ml) were charged into a three-necked round-bottomed flask fitted with a mechanical stirrer, a thermometer and a nitrogen inlet. The mixture was stirred vigorously at 120°C for 3 h, then the mixture was heated to 166°C and kept for 5 h under nitrogen atmosphere. After the reactor was cooled to room temperature, the reaction solution was poured into water. The resulting solid was filtered, washed with hot water and methanol, dried and recrystallized from a mixture of dimethyl formamide and water (3:1 v/v) to get a yellow powder. Light yellow crystals suitable for X-ray analysis were obtained by slow evaporation of a formamide/water (3:1 v/v) solution at 60°C.

### Refinement

The H atoms bound to the N atom were found in a difference Fourier map and refined freely. All other H atoms were positioned geometrically and refined using a riding model, with C—H = 0.93 Å and *U*_iso_(H) = 1.2*U*_eq_ (C).

### Crystal data

**Table:**

C_25_H_20_N_2_OS_2_	*F*(000) = 896
*M**_r_* = 428.55	*D*_x_ = 1.282 Mg m^−^^3^
Monoclinic, *C*2/*c*	Mo *K*α radiation, λ = 0.71073 Å
Hall symbol: -C 2yc	Cell parameters from 31 reflections
*a* = 18.945 (3) Å	θ = 4.3–9.4°
*b* = 6.025 (2) Å	µ = 0.26 mm^−^^1^
*c* = 20.793 (5) Å	*T* = 292 K
β = 110.64 (4)°	Block, yellow
*V* = 2221.1 (11) Å^3^	0.52 × 0.48 × 0.42 mm
*Z* = 4	

### Data collection

**Table:**

Enraf–Nonius CAD-4 diffractometer	1441 reflections with *I* > 2σ(*I*)
Radiation source: fine-focus sealed tube	*R*_int_ = 0.010
graphite	θ_max_ = 25.5°, θ_min_ = 2.1°
ω–2θ scans	*h* = −22→21
Absorption correction: for a sphere (*WinGX*; Farrugia, 1999)	*k* = 0→7
*T*_min_ = 0.877, *T*_max_ = 0.899	*l* = −18→24
2261 measured reflections	3 standard reflections every 150 reflections
1990 independent reflections	intensity decay: 2.4%

### Refinement

**Table:**

Refinement on *F*^2^	Primary atom site location: structure-invariant direct methods
Least-squares matrix: full	Secondary atom site location: difference Fourier map
*R*[*F*^2^ > 2σ(*F*^2^)] = 0.050	Hydrogen site location: mixed
*wR*(*F*^2^) = 0.146	H atoms treated by a mixture of independent and constrained refinement
*S* = 1.05	*w* = 1/[σ^2^(*F*_o_^2^) + (0.0979*P*)^2^ + 0.2492*P*] where *P* = (*F*_o_^2^ + 2*F*_c_^2^)/3
1990 reflections	(Δ/σ)_max_ = 0.001
145 parameters	Δρ_max_ = 0.22 e Å^−^^3^
0 restraints	Δρ_min_ = −0.30 e Å^−^^3^

### Special details

**Table:**

Geometry. All e.s.d.'s (except the e.s.d. in the dihedral angle between two l.s. planes) are estimated using the full covariance matrix. The cell e.s.d.'s are taken into account individually in the estimation of e.s.d.'s in distances, angles and torsion angles; correlations between e.s.d.'s in cell parameters are only used when they are defined by crystal symmetry. An approximate (isotropic) treatment of cell e.s.d.'s is used for estimating e.s.d.'s involving l.s. planes.
Refinement. Refinement of *F*^2^ against ALL reflections. The weighted *R*-factor *wR* and goodness of fit *S* are based on *F*^2^, conventional *R*-factors *R* are based on *F*, with *F* set to zero for negative *F*^2^. The threshold expression of *F*^2^ > 2σ(*F*^2^) is used only for calculating *R*-factors(gt) *etc*. and is not relevant to the choice of reflections for refinement. *R*-factors based on *F*^2^ are statistically about twice as large as those based on *F*, and *R*- factors based on ALL data will be even larger.

### Fractional atomic coordinates and isotropic or equivalent isotropic displacement parameters (Å^2^)

**Table:**

	*x*	*y*	*z*	*U*_iso_*/*U*_eq_	
S1	0.59611 (4)	0.88028 (12)	0.52196 (3)	0.0591 (3)	
O1	0.5000	0.1894 (4)	0.2500	0.0638 (7)	
N1	0.8241 (2)	0.3345 (7)	0.73370 (17)	0.0954 (11)	
HN1	0.811 (2)	0.224 (7)	0.7515 (19)	0.096 (13)*	
HN2	0.8651 (18)	0.371 (5)	0.7407 (16)	0.071 (11)*	
C1	0.5000	0.3937 (5)	0.2500	0.0397 (7)	
C2	0.52388 (11)	0.5146 (3)	0.31641 (10)	0.0364 (5)	
C3	0.57458 (12)	0.4134 (4)	0.37505 (11)	0.0427 (5)	
H3	0.5933	0.2731	0.3714	0.051*	
C4	0.59739 (12)	0.5168 (4)	0.43805 (11)	0.0437 (5)	
H4	0.6312	0.4466	0.4764	0.052*	
C5	0.56977 (12)	0.7271 (4)	0.44448 (11)	0.0409 (5)	
C6	0.51783 (12)	0.8281 (4)	0.38664 (10)	0.0396 (5)	
H6	0.4979	0.9662	0.3907	0.048*	
C7	0.49596 (11)	0.7241 (3)	0.32367 (10)	0.0371 (5)	
H7	0.4621	0.7944	0.2854	0.045*	
C8	0.66367 (12)	0.7082 (4)	0.58244 (11)	0.0484 (6)	
C9	0.73981 (14)	0.7565 (5)	0.60170 (13)	0.0606 (7)	
H9	0.7555	0.8750	0.5814	0.073*	
C10	0.79254 (14)	0.6301 (5)	0.65085 (14)	0.0650 (8)	
H10	0.8435	0.6644	0.6631	0.078*	
C11	0.77125 (14)	0.4541 (5)	0.68221 (12)	0.0580 (7)	
C12	0.69484 (15)	0.4026 (5)	0.66200 (13)	0.0634 (7)	
H12	0.6794	0.2819	0.6816	0.076*	
C13	0.64197 (13)	0.5290 (5)	0.61320 (12)	0.0567 (7)	
H13	0.5911	0.4940	0.6007	0.068*	

### Atomic displacement parameters (Å^2^)

**Table:**

	*U*^11^	*U*^22^	*U*^33^	*U*^12^	*U*^13^	*U*^23^
S1	0.0615 (5)	0.0678 (5)	0.0395 (4)	0.0091 (3)	0.0073 (3)	−0.0110 (3)
O1	0.0882 (19)	0.0344 (13)	0.0499 (14)	0.000	0.0009 (13)	0.000
N1	0.0612 (19)	0.134 (3)	0.084 (2)	0.0128 (19)	0.0163 (16)	0.042 (2)
C1	0.0395 (16)	0.0318 (16)	0.0397 (16)	0.000	0.0038 (13)	0.000
C2	0.0376 (11)	0.0363 (11)	0.0336 (10)	−0.0016 (9)	0.0103 (8)	0.0045 (8)
C3	0.0456 (12)	0.0357 (12)	0.0413 (12)	0.0045 (9)	0.0084 (10)	0.0046 (9)
C4	0.0408 (12)	0.0475 (13)	0.0364 (11)	0.0030 (10)	0.0058 (9)	0.0049 (10)
C5	0.0375 (11)	0.0475 (13)	0.0379 (11)	−0.0045 (10)	0.0137 (9)	−0.0013 (9)
C6	0.0395 (11)	0.0413 (12)	0.0401 (11)	0.0021 (9)	0.0166 (10)	0.0012 (9)
C7	0.0350 (10)	0.0383 (11)	0.0366 (11)	0.0017 (9)	0.0109 (9)	0.0039 (9)
C8	0.0412 (13)	0.0668 (16)	0.0348 (11)	−0.0065 (11)	0.0102 (10)	−0.0071 (11)
C9	0.0500 (14)	0.0760 (18)	0.0540 (15)	−0.0172 (13)	0.0164 (12)	0.0067 (13)
C10	0.0364 (13)	0.092 (2)	0.0602 (16)	−0.0089 (13)	0.0092 (12)	0.0043 (15)
C11	0.0501 (15)	0.0776 (17)	0.0433 (13)	0.0015 (13)	0.0126 (11)	0.0023 (12)
C12	0.0585 (16)	0.079 (2)	0.0537 (15)	−0.0130 (14)	0.0206 (13)	0.0093 (13)
C13	0.0380 (12)	0.0843 (19)	0.0454 (13)	−0.0144 (12)	0.0115 (10)	−0.0014 (13)

### Geometric parameters (Å, °)

**Table:**

S1—C5	1.769 (2)	C5—C6	1.397 (3)
S1—C8	1.777 (3)	C6—C7	1.377 (3)
O1—C1	1.231 (3)	C6—H6	0.9300
N1—C11	1.383 (4)	C7—H7	0.9300
N1—HN1	0.84 (4)	C8—C9	1.385 (3)
N1—HN2	0.77 (3)	C8—C13	1.389 (4)
C1—C2	1.484 (2)	C9—C10	1.380 (4)
C1—C2^i^	1.484 (2)	C9—H9	0.9300
C2—C7	1.397 (3)	C10—C11	1.377 (4)
C2—C3	1.398 (3)	C10—H10	0.9300
C3—C4	1.375 (3)	C11—C12	1.393 (4)
C3—H3	0.9300	C12—C13	1.377 (4)
C4—C5	1.395 (3)	C12—H12	0.9300
C4—H4	0.9300	C13—H13	0.9300
			
C5—S1—C8	103.99 (12)	C6—C7—C2	121.02 (19)
C11—N1—HN1	121 (2)	C6—C7—H7	119.5
C11—N1—HN2	114 (2)	C2—C7—H7	119.5
HN1—N1—HN2	125 (3)	C9—C8—C13	118.5 (2)
O1—C1—C2	119.39 (12)	C9—C8—S1	119.9 (2)
O1—C1—C2^i^	119.39 (12)	C13—C8—S1	121.52 (17)
C2—C1—C2^i^	121.2 (2)	C10—C9—C8	120.4 (2)
C7—C2—C3	118.00 (18)	C10—C9—H9	119.8
C7—C2—C1	122.75 (18)	C8—C9—H9	119.8
C3—C2—C1	119.21 (19)	C11—C10—C9	121.3 (2)
C4—C3—C2	121.4 (2)	C11—C10—H10	119.4
C4—C3—H3	119.3	C9—C10—H10	119.4
C2—C3—H3	119.3	C10—C11—N1	120.9 (3)
C3—C4—C5	120.0 (2)	C10—C11—C12	118.4 (2)
C3—C4—H4	120.0	N1—C11—C12	120.7 (3)
C5—C4—H4	120.0	C13—C12—C11	120.5 (3)
C4—C5—C6	119.15 (19)	C13—C12—H12	119.7
C4—C5—S1	124.48 (16)	C11—C12—H12	119.7
C6—C5—S1	116.37 (17)	C12—C13—C8	120.8 (2)
C7—C6—C5	120.3 (2)	C12—C13—H13	119.6
C7—C6—H6	119.8	C8—C13—H13	119.6
C5—C6—H6	119.8		
			
O1—C1—C2—C7	−150.51 (15)	C3—C2—C7—C6	0.2 (3)
C2^i^—C1—C2—C7	29.49 (15)	C1—C2—C7—C6	178.04 (18)
O1—C1—C2—C3	27.3 (2)	C5—S1—C8—C9	102.6 (2)
C2^i^—C1—C2—C3	−152.7 (2)	C5—S1—C8—C13	−79.8 (2)
C7—C2—C3—C4	−0.8 (3)	C13—C8—C9—C10	−0.6 (4)
C1—C2—C3—C4	−178.80 (18)	S1—C8—C9—C10	177.1 (2)
C2—C3—C4—C5	0.1 (3)	C8—C9—C10—C11	−0.3 (4)
C3—C4—C5—C6	1.3 (3)	C9—C10—C11—N1	−177.0 (3)
C3—C4—C5—S1	−178.56 (17)	C9—C10—C11—C12	1.5 (4)
C8—S1—C5—C4	1.7 (2)	C10—C11—C12—C13	−1.8 (4)
C8—S1—C5—C6	−178.18 (16)	N1—C11—C12—C13	176.7 (3)
C4—C5—C6—C7	−2.0 (3)	C11—C12—C13—C8	0.9 (4)
S1—C5—C6—C7	177.89 (16)	C9—C8—C13—C12	0.3 (4)
C5—C6—C7—C2	1.3 (3)	S1—C8—C13—C12	−177.4 (2)

Symmetry codes: (i) −*x*+1, *y*, −*z*+1/2.

### Hydrogen-bond geometry (Å, °)

**Table:**

*D*—H···*A*	*D*—H	H···*A*	*D*···*A*	*D*—H···*A*
N1—HN2···O1^ii^	0.77 (3)	2.52 (3)	3.231 (4)	154 (3)

Symmetry codes: (ii) −*x*+3/2, −*y*+1/2, −*z*+1.
